# Validity and Calibration of the Youth Activity Profile

**DOI:** 10.1371/journal.pone.0143949

**Published:** 2015-12-02

**Authors:** Pedro F. Saint-Maurice, Gregory J. Welk

**Affiliations:** Department of Kinesiology, Iowa State University, Ames, Iowa, United States of America; Pulmonary Research Institute at LungClinic Grosshansdorf, GERMANY

## Abstract

**Purpose:**

The purpose of this study was to calibrate and cross-validate the Youth Activity Profile (YAP), a self-report tool designed to capture physical activity (PA) and sedentary behaviors (SB) in youth.

**Methods:**

Eight schools in the Midwest part of the U.S. were involved and a total of 291 participants from grades 4–12 agreed to wear an accelerometer (SWA Armband) and complete the YAP in two separate weeks (5–7 days apart). Individual YAP items capture PA behavior during specific segments of the week and these items were combined with temporally matched estimates of moderate-to-vigorous PA (MVPA) and sedentary time from the SWA to enable calibration. Quantile regression procedures yielded YAP prediction algorithms that estimated MVPA at School, MVPA at Out-of-School, MVPA on Weekend, as well as time spent in SB. The YAP estimates of time spent in MVPA and SB were cross-validated using Pearson product correlations and limits of agreement, as indicative of individual error and, equivalence testing techniques as indicative of group-level error.

**Result:**

Following calibration, the correlations between YAP and SWA estimates of MVPA were low to moderate (r_range_ = .19 to .58) and individual-level YAP estimates of MVPA ranged from -134.9% to +110.0% of SWA MVPA values. Differences between aggregated YAP and SWA MVPA ranged from -3.4 to 21.7 minutes of MVPA at the group-level and predicted YAP MVPA estimates were within 15%, 20%, and 30%, of values from the SWA for the School, Out-of-School, and Weekend time periods, respectively. Estimates of time spent in SB were highly correlated with each other (r = .75). The individual estimates of SB ranged from -54.0% to +44.0% of SWA sedentary time, and the aggregated group-level estimates differed by 49.7 minutes (within 10% of the SWA aggregated estimates).

**Conclusions:**

This study provides preliminary evidence that the calibration procedures enabled the YAP to provide estimates of MVPA and SB that approximated values from an objective monitor. The YAP provides a simple, low-cost and educationally sound method to accurately estimate children’s MVPA and SB at the group level.

## Introduction

The development of accurate assessments of physical activity (PA) is an important research priority since it impacts the ability to advance research in a number of areas. Self-report instruments are clearly the most efficient and cost effective tool but a significant concern (and limitation) is the inherent subjectivity and perceived inaccuracy when compared with objective monitors. However, the use of measurement error models and calibration equations offer potential for correcting bias and error in self-report measures [1,2]. Calibration methods have been routinely used to improve the utility/accuracy of objective monitoring devices [3,4,5,6,7,8,9]. These calibration procedures have enabled raw movement counts to be converted into more meaningful units such as energy expenditure (EE) or time spent in physical activity (PA). This same approach offers potential to improve the utility of self-report measures. In this case, self-report measures can be calibrated against objective monitors to produce more accurate estimates of EE or time spent in PA.

Calibration offers potential for all segments of the population but it may be particularly advantageous for assessing PA in youth. For example, a key need in youth research and public health surveillance is to develop assessment tools that can be used in school settings to capture data on large samples of children. Numerous studies have questioned the ability of children to accurately complete self-report forms but the limitation may be due to inability of current self-report tools to capture the sporadic and intermittent nature of children’s activity. This is evidenced by the fact that the various self-report tools for youth have consistently yielded low to moderate correlations with objective tools [10]. We recently demonstrated the potential utility of calibrating the Physical Activity Questionnaire (PAQ) a commonly used physical activity questionnaire for youth [11]. We found that simple calibration enabled the PAQ to provide a reasonable group-level prediction of time spent in moderate-to-vigorous PA (MVPA). We also found that estimates obtained from the calibration model developed for the PAQ could be used to classify youth meeting the PA guidelines. Classifications approximated the distributions of the proportion of youth meeting PA guidelines obtained from the accelerometer (AUC = 0.79) [11].

While the results supported the utility of the PAQ calibration, there are some inherent limitations of the PAQ for use in evaluating youth activity patterns. One limitation is that the PAQ does not capture some important windows of the day where activity is likely to occur (e.g., before school, commuting to school). Therefore, predicted minutes of activity obtained from the PAQ would most likely underestimate daily activity. The PAQ also captures total weekend activity with a single item but it is likely that activity patterns vary considerably between Saturday and Sunday for most youth. Other items on the PAQ overlap and ask about activity during similar time frames. For example, question 8 and question 9 of the PAQ-C ask about overall levels of activity during the last 7 days. These are useful for evaluating overall patterns but they are redundant with other items and therefore not of much value for calibration purposes. The last, and perhaps most significant, limitation of the PAQ is that it does not include any measure of sedentary behaviors (SB). The consensus in the literature is that SB is distinct from PA and merits independent evaluation [12,13,14,15]. The PAQ has considerable utility as a self-report measure of PA but it is not ideally suited for calibration purposes. To address these limitations, we developed a new self-report tool called the Youth Activity Profile (YAP) and have initiated a systematic and ongoing calibration study to continually improve the calibration of this tool [16]. This study builds on our previous calibration work with the PAQ but incorporates a couple of study design improvements to improve the quality and utility of the calibrated data. Objective data on physical activity behavior were obtained from the SWA activity monitor instead of the Actigraph. Data were also collected across seasons (also balanced by age) in order to capture more variability in activity patterns. Therefore, the purpose of this study was to report the preliminary calibration results for estimating time spent in PA and SB in youth with the YAP.

## Methods

### Participants

A total of 29 schools in Iowa were contacted to participate in the Youth Physical Activity Measurement Study (YPAMS). Eight schools (2 elementary, 3 middle, and 3 high schools) agreed to participate and 625 students from 4^th^ to 12th grade were informed about the study.

### Ethics Statement

This study was approved by the Institutional Review Board at Iowa State University. All students received an enrollment package containing both consent and assent forms. The consent form for all participants was required to be signed by the parent or legal guardian and participants were also required to provide written assent for participation.

### Instruments

#### SenseWear Armband Pro3

The SenseWear Armband Pro3 (SWA) (BodyMedia, Inc., Pittsburgh, PA) is a wireless pattern-recognition device that integrates motion sensor data with a variety of heat-related sensors, and demographic variables to estimate the energy expenditure [17]. This monitor has been tested in children and has been shown to provide accurate estimates of PA and EE in this population [18,19]. Results from a doubly labeled water study demonstrated error rates of less than 11% [20]. An additional advantage of the SWA for field-based research is that it automatically detects non-wear time. The SWA was initialized with 1-min epochs and data were downloaded using InnerView v6.1 software.

#### Youth Activity Profile

The Youth Activity Profile (YAP) is a 15-item self-report instrument designed to capture physical activity and sedentary behavior in youth [16]. It was based conceptually on the time based structure of the established Physical Activity Questionnaire for Children (PAQ-C) and the related tool for Adolescents (PAQ-A) [21,22,23,24]. The YAP was designed to be a self-administered 7-day (previous week) recall questionnaire suitable for use in children grades 4–12. The YAP includes a total of 15 items divided into three sections: 1) Activity at School, 2) Activity Out-of-School, and 3) Sedentary Habits. Items in the At School section capture participation in MVPA during 5 specific windows of the day (transportation to and from school, as well as activity during physical education, lunch, and recess). Items in the Out-of-School section include activity before school, activity right after-school, activity during the evening, and activity in each weekend day (Saturday and Sunday). Sedentary items, ask about time spent watching TV, playing videogames, using the computer, using a cell phone, and also include an overall sedentary time item. The final version of the YAP was subjected to pilot testing and cognitive interviews were performed with similarly aged participants in order to refine the final items. In this study, we used the paper version of the Youth Activity Profile (YAP); however, an online version of the YAP has also been developed to facilitate use in schools [16]. The print version, used in this study, takes approximately 7–10 minutes to be completed.

### Design and Procedures

Data were collected during a full academic year (Fall and Spring semesters) and counterbalanced among elementary, middle and high school participants. Data were collected in classroom groups and each group was assessed twice (two weeks) with a 5–7 day interval. At the first visit of data collection, participants were provided with instructions on how to wear the SWA monitor and were given an activity log and asked to record activities during non-wear periods.

The following week, they returned the monitor and were provided with a copy of the YAP to complete. Upon completion of the YAP, participants were asked to visit with a staff member and YAP answers were examined individually, using specific recall probes. The probes increased the time of completion of the YAP but helped to ensure that students understood the questions and were responding based on their actual behavior and not typical behavior or interests. These probes were specifically developed for the calibration and are not part of the YAP nor are recommended for field applications when using this tool.

### Data Processing

The SWA accelerometer data were downloaded from the InnerView software v6.1, exported in 1 minute intervals, and processed using SAS v9.2. Data from the YAP were temporally matched with accelerometer data processed from the same respective windows of time. Accelerometer data were first screened for abnormal/missing energy expenditure values and processed using comprehensive data reduction procedures. Participants were given an activity log and asked to record activities during non-wear periods. These activities were imputed in the raw data set using recommended procedures [25]. A matching MET value was obtained from the Compendium of Physical Activities and available literature [26,27].

Minute-by-minute predicted METs generated by the SWA were classified into minutes of activity and categorized as MVPA if ≥ 4.0 METs based on convention with youth. Sedentary time was defined as periods with METs <2.0 since this cutpoint also provides a more accurate classifications of sedentary activities than the adult value of 1.5 METS [28].

The Youth Activity Profile scores in each item were first examined individually and later (after calibration and conversion to minutes of activity/sedentary time), summed to reflect activity levels during school and home environment. Questions 11 through 15 (Sedentary section) were averaged to create a composite score and matched with accelerometer sedentary time recorded during afterschool and evening windows.

### Data Analyses

A key need in the analyses was to link the reported data to temporally matched data from the SWA. Therefore, activity segments of the week were defined as the unit of observation in the study and defined based on individualized schedules provided by schools [16]. A total of 10 specific windows were established corresponding to the specific periods of time captured o the YAP (i.e., transportation to school, recess, lunch, PE, transportation from school, afterschool, evening, before school, Saturday and Sunday). A separate weekday estimate of total weekly SB time was used since the YAP items for SB emphasized total time rather than amounts in specific segments.

There were two phases of analyses, a calibration phase and a cross-validation phase. In the calibration phase, the dependent variables were the daily percent time spent in MVPA and the percent time in sedentary behavior while age, gender, and the matching YAP item, were defined as independent variables. The use of percent time in activity was a preferred outcome measure since it enables better control over the variations in the lengths of individual segments. In the cross validation analyses, the prediction algorithms for individual segments were used to produce corresponding estimates of time spent in MVPA. These were then aggregated to produce separate estimates of MVPA for In School, Out-of-School time periods, as well as total weekly SB time. Details on the calibration and cross-validation phase are described below.

#### Calibration Analyses

Data from week one were used in this phase. If week one data was missing, then data from week two were used. This approach was preferred in order to maximize our sample size and improve the robustness of the algorithms obtained. Regression models were generated for each window using quantile regression, set at the 50^th^ quantile (median) and the β-weights were tested using the Wald test. Confidence intervals were obtained using the bootstrap method and significance was set at an alpha of .05. Because some non-linear relations were expected, these were handled using spline regression procedures.

#### Cross-Validation Analyses

The final regression models were used to predict daily percent time in MVPA/sedentary time using week two activity scores. Data were screened for outliers similarly to the week one data and predicted item MVPA values were aggregated to reflect MVPA at School, MVPA at Out-of-School during weekdays, MVPA at Out-of-School during the weekend, and Out-of-School sedentary time (S1 File). The validity of each YAP section estimate (including a separate section for Weekend) and total MVPA was examined by computation of respective individual- and group-level error. Individual error reflects to what extent predicted activity (i.e., minutes of MVPA obtained from the YAP) can replicate the measured activity (i.e., minutes of MVPA obtained from the SWA) in a single child. Group error, in contrast, reflects the average difference between predicted and measured minutes of activity obtained from a group of youth. Individual- and group-level error were computed using Limits of Agreement [29] and bioequivalence testing, respectively.

Bioequivalence was determined using two one-sided paired t-tests (90% confidence interval, or 5% for each lower and upper limit) for the mean difference between SWA and YAP MVPA [30]. Equivalence was supported if the confidence interval for the mean difference was within 10% of the SWA minutes of MVPA. The selection of a 10% equivalence region was arbitrarily defined so less conservative equivalence regions were also tested in case that equivalence was not supported for the 10% level. These additional tests were conducted using 5% increments in the region of equivalence (i.e., 15%, 20%, and 25%). This procedure was selected in order to provide a clear estimation of the accuracy of the YAP. All analyses were done using SAS v9.2.

## Results

There were 291 participants (135 elementary, 67 middle, and 89 high school participants; 128 boys and 163 girls) with valid activity estimates in at least one of the segmented activity windows (S2 File). Additionally, there were 147 participants with valid Non-Winter data and 144 participants that had their data collected in the Winter. The overall distribution of age groups and gender between the two seasons was approximately balanced. On average participants wore the accelerometer for 8.8 hours a day during a week day (528.4 minutes) and for 12.8 hours during a weekend day (768.7 minutes) (Table 1).

**Table 1:** Descriptive information and accelerometer wear time, by age group.

	N	Age	Height	Weight	BMI_PCT_ ^1^	Wear Time Week^2^	Wear Time Weekend^3^	Expected Week^4^	Expected Weekend^5^
**Elementary School**	**135**	**9.7±1.0**	**143.0±7.7**	**37.9±10.4**	**60.1±28.0**	**529.4±7.0**	**759.4±49.9**	**615.9±35.2**	**900**
**Middle School**	**67**	**11.7±0.8**	**155.4±6.5**	**49.7±13.6**	**61.9±31.5**	**513.3±6.2**	**766.3±36.6**	**582.0±27.2**	**900**
**High School**	**89**	**15.7±1.2**	**167.3±10.7**	**64.1±12.7**	**66.3±26.1**	**542.8±17.4**	**780.3±63.1**	**616.4±69.6**	**900**

^1^ BMI percentiles; computed as recommended by the Centers for Disease Control and Prevention

^2^ Average time (in minutes) per week day that participants wore the accelerometer

^3^ Average time (in minutes) per weekend day that participants wore the accelerometer

^4^ Average total duration of week day in minutes; note that only data from specific windows of activity during the day were used for analysis; also note that schedules were not similar among participants therefore the total duration expected varied to some degree.

^5^ Total duration of weekend day in minutes; this value was equal to 900 minutes and didn’t have any variability since weekend days were set for 15 hours duration (7:00AM to 10:00PM) and equal among all participants.

### Calibration

A total of 291 observations (most from week 1) were used in the calibration phase. Results are provided separately for each segment of the YAP.

#### Section I: School Activity

All School activity items were significant predictors of daily percent time in MVPA except the Lunch item (β = 1.38 ± 1.85, p = .46). However, the intercept for this item was tested (with age and gender set at their average score and YAP lunch set at a median score of 3) to determine if level of activity during this time period was different than 0. The intercept estimate was equal to 17.4 ± 3.1 and deemed significant (p < .001) indicating that there was some residual activity accumulated during lunch. Residual activity was therefore predicted setting the lunch YAP score at a median value of 3. The β-weight associated with reported scores on the Physical Education (PE) item was borderline significant (β = 8.92 ± 4.88, p = .07). Upon visualization of the data, the recess and PE item were not linearly related with respective SWA scores. These specific algorithms were adjusted using spline regression to account for this non-linear relation and by incorporating a plateau factor in each of these models.

#### Section II: Out-of-School Activity

Items from the Out-of-School section were similarly explored and used to predict daily %MVPA during respective out-of-school segments. All of these were significant predictors of activity except the Before School (β = -2.83 ± 1.68, p = .09) and Sunday items (β = 0.76 ± 0.69, p = .27). Additional test of the intercept for the Sunday calibration algorithm indicated that there was significant residual activity accumulated during this period (Intercept = 7.8 ± 1.1, p < .001) and therefore it was decided to set the β-weight for this item at a median score of 3. Similarly to the recess and PE school items, both the evening and Saturday items were adjusted for non-linear relations found for this activity segments.

#### Section III: Home Sedentary

In this section, the daily percent sedentary time during after-school and evening periods were used as the outcome variable. The videogame item was not related with percent sedentary time (r_s_ (190) = .02, p = .78) therefore, a composite YAP score for this section did not include this item. The YAP composite score (β = 9.88 ± 2.40, p < .001) was a significant predictor of recorded percent time in sedentary (S3 & S4 Files).

### Cross-Validation

There were 161 participants with valid replicate data on at least one segmented window of activity. Similarly to the calibration phase, results are provided separately for each of the segments of the YAP.

#### Section I: School Activity

**Fig 1:**
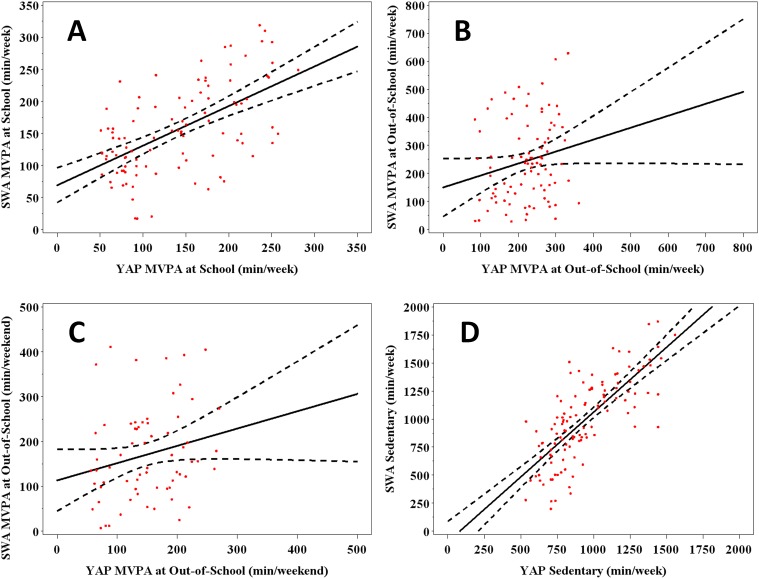
Relation between measured and predicted minutes of moderate-to-vigorous PA during school (A), out-of-school (week—B) and out-of-school (weekend—C). Panel D illustrates the relation between measured and predicted minutes of sedentary activity. The line of best fit (solid black line) and respective 95% confidence interval (dashed black line) are provided.

**Fig 2:**
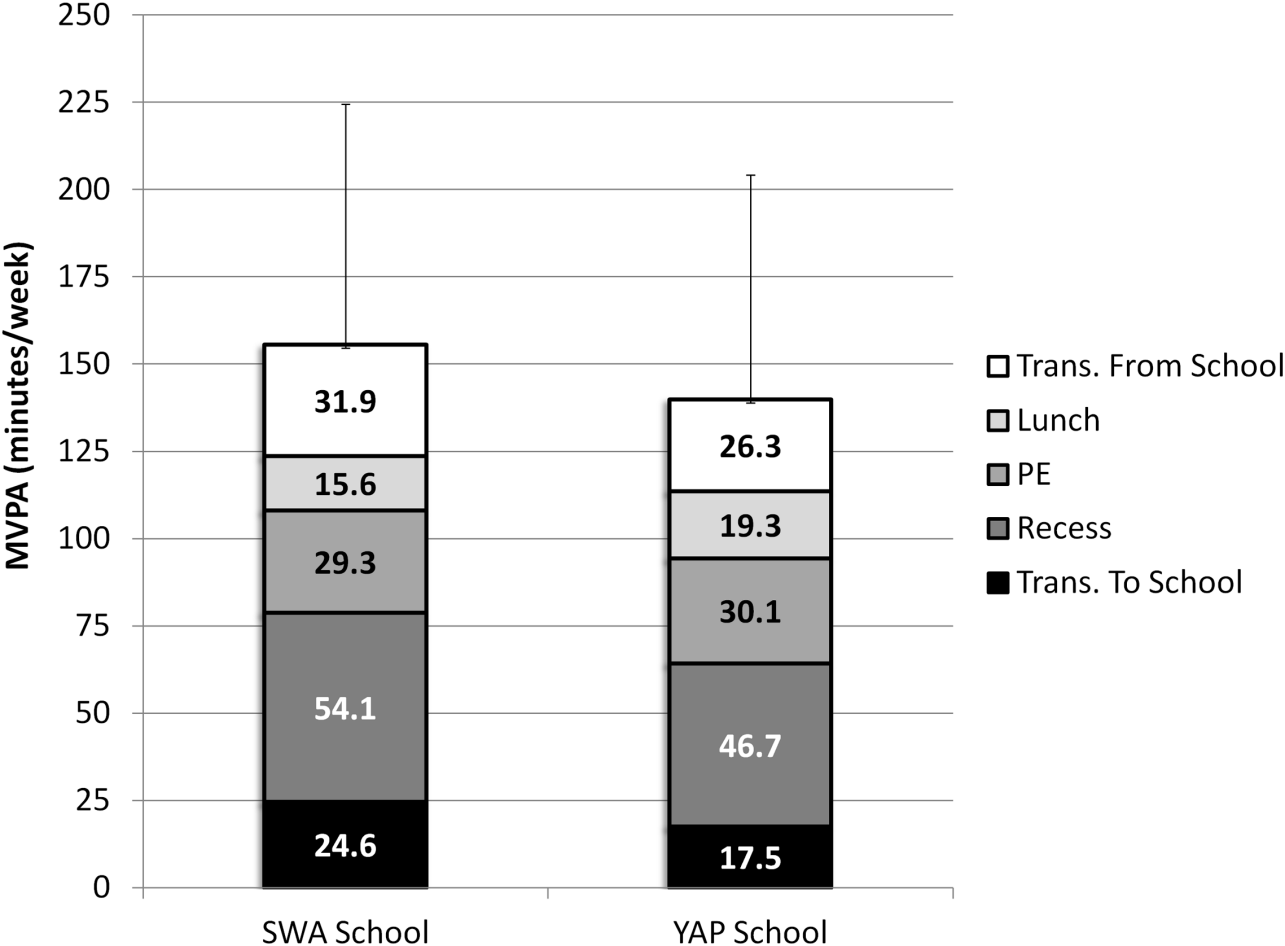
Average estimates of recoded and predicted MVPA and respective standard deviation for weekly activity at school (segmented by window of activity).

The YAP and SWA school estimates were moderately correlated (r (99) = .58, p < .001) (Fig 1 –Panel A) and individual–level error ranged between -87% and +67% of SWA activity measured during this time (Table 2). Once aggregated into weekly estimates, recorded activity was equal to 155.5 ± 68.9 minutes of MVPA while predicted MVPA for this same time segment was equal to 139.9 ± 64.3 minutes (a difference of -15.6 ± 6.2 minutes). The 90% confidence interval for the mean difference (-25.8 to -5.3 minutes of MVPA) fell outside the 10% equivalence region (-15.5 to +15.5 minutes) so estimates of activity at school were not considered equivalent at a 10% region. Follow-up examinations for agreement defined equivalence at 20% (-31.1 to +31.1 minutes) (Table 2). Fig 2 provides an illustration of the average measured and estimated MVPA obtained at each school segment and their contribution to total activity time at school.

**Table 2:** Summary of individual- and group-level agreement.

Segment	Mean diff ± Std^1^	Individual Error^2^	Group Error^3^
**Activity time**			
**School**	**-15.6 ± 61.4**	**-87.0% to +67.0%**	**15%**
**Out-of-School Week**	**3.4 ± 157.3**	**-112.4% to +110.0%**	**20%**
**Out-of-School Weekend**	**-21.7 ± 107.0**	**-134.9% to +109.6%**	**30%**
**Sedentary time**			
**Out-of-School Week**	**-49.7 ± 248.8**	**-54% to +44%**	**10%**

^1^ Mean Difference (YAP—SWA) ± Standard Deviation of Mean Difference

^2^ Computed as (Mean Difference ± (1.96 x Standard Deviation of Mean Difference)/Mean SWA value) x 100

^3^ Determined using bioequivalence testing with two one-sided paired t-tests and using 90% confidence intervals.

#### Section II: Out-of-School Activity—Weekday

Out-of-school estimates were aggregated to provide separate indicators of weekly activity (computed as: Before School MVPA+ After-School MVPA+ Evening MVPA) outside of the school setting. Recorded and predicted activity scores were not significantly correlated (r (89) = .19, p = .07) (Fig 1 –Panel B) and estimated YAP activity ranged from -112.4% to +110% of measured activity, suggesting poor individual-level agreement (Table 2).

**Fig 3:**
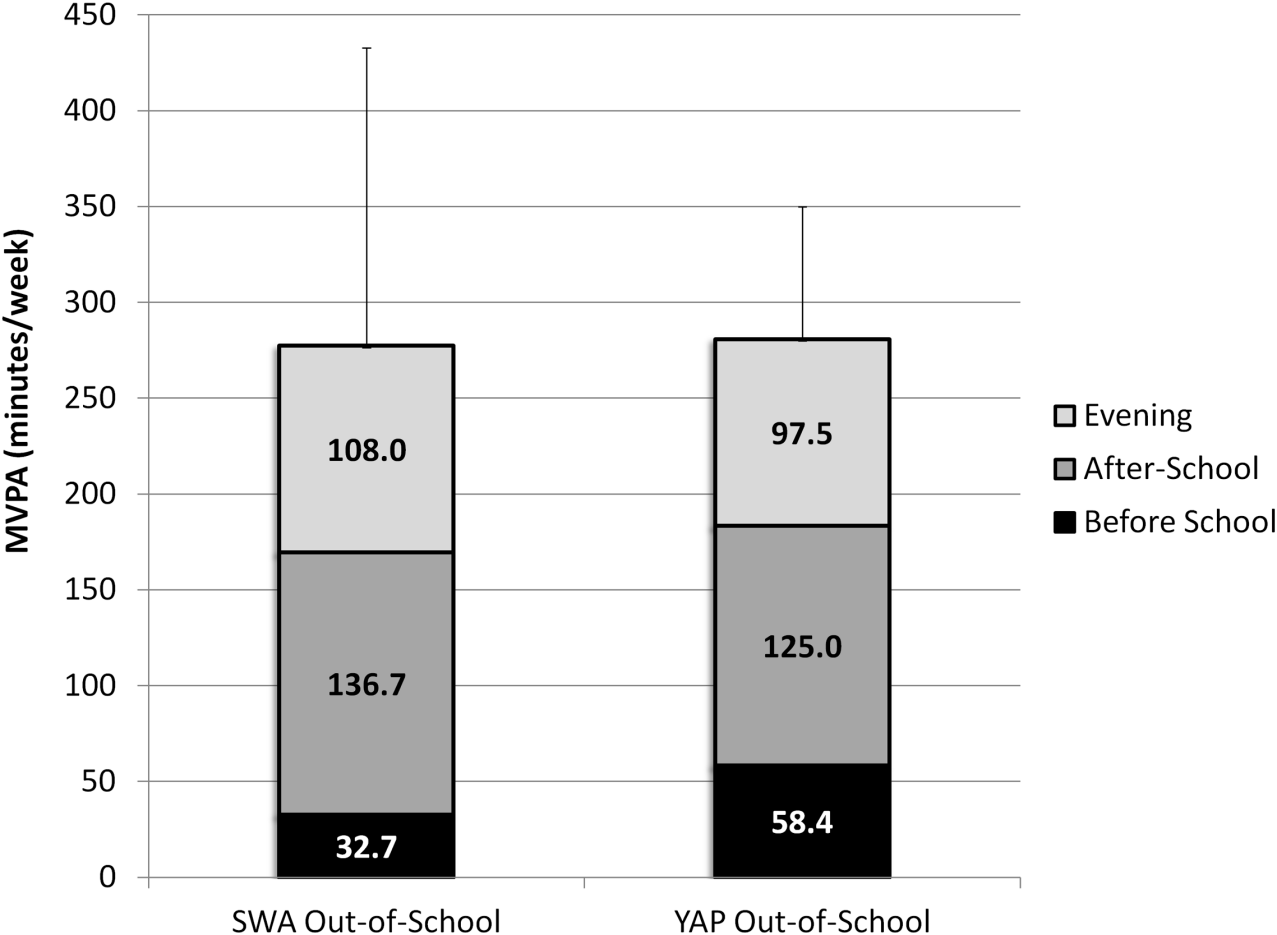
Average estimates and respective standard deviations of recorded and predicted MVPA for weekly out-of-school activity (segmented by window of activity).

Aggregated SWA minutes of MVPA resulted in 277.4 ± 155.3 minutes of MVPA while predicted MVPA from the YAP was 280.8 ± 69.1 minutes (difference = 3.4 ± 16.6 minutes). The 90% confidence interval for the mean difference was -24.2 and +31.0 minutes and was outside the 10% equivalence region (-27.7 to +27.7 minutes). Therefore, estimates were not considered equivalent. Additional analyses showed that the measures were equivalent at the 15% level (-41.6 to +41.6 minutes) (Table 2). Recorded and measured estimates of MVPA for each out-of-school segment are provided in Fig 3.

#### Section II: Out-of-School Activity—Weekend

Out-of-school activity was also aggregated into weekend activity estimates. These two estimates were not significantly correlated (r (66) = .22, p = .08) (Fig 1 –Panel C) and the two items predicted individual’s activity with an error ranging between -134.9% and +109.6% of SWA values (Table 2).

**Fig 4:**
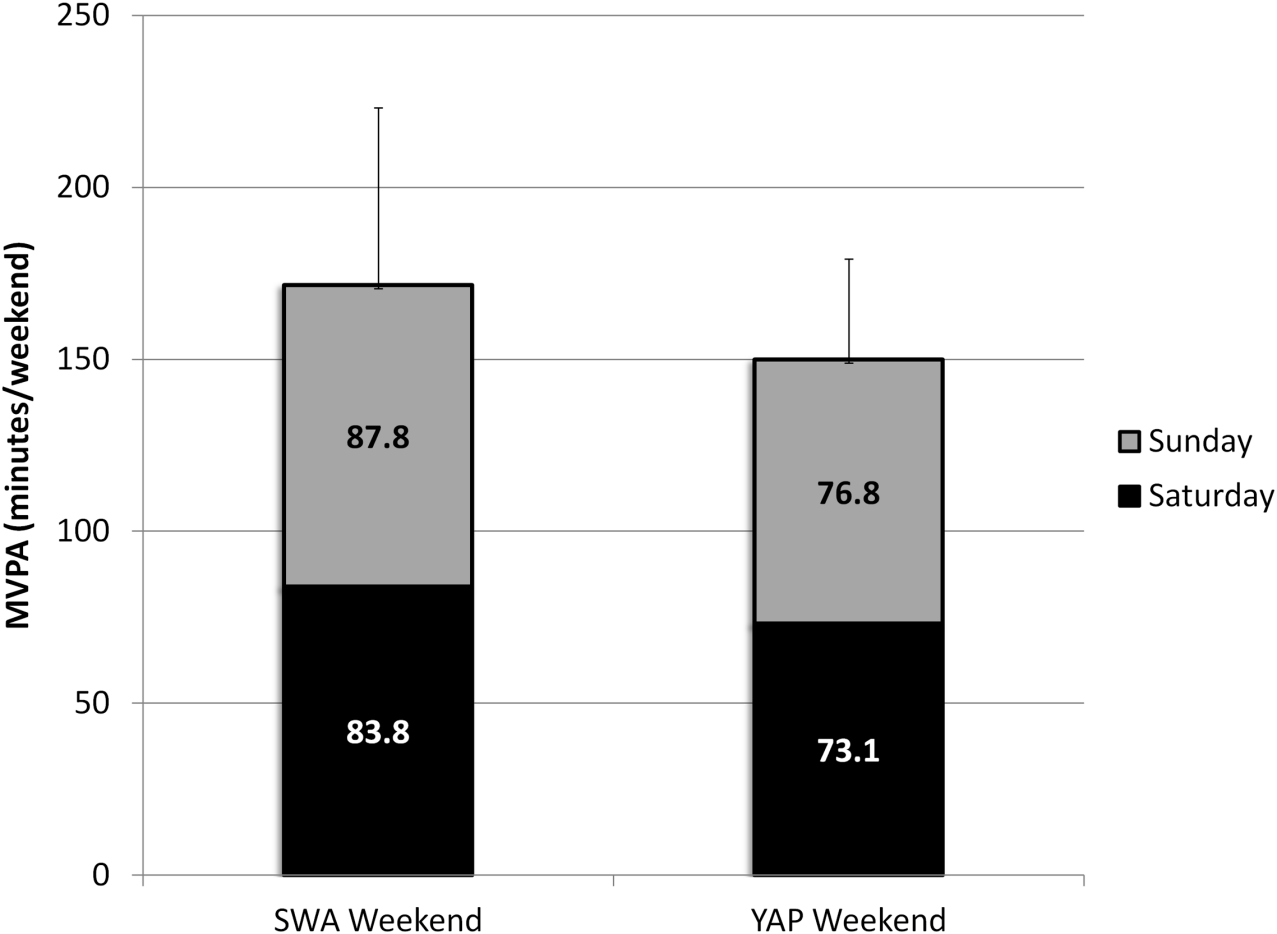
Average estimates and respective standard deviations of recorded and predicted MVPA for weekend out-of-school activity (segmented by window of activity).

Average SWA MVPA for this time segment was 171.6 ± 103.3 while predicted MVPA resulted in 149.9 ± 58.7 minutes, resulting in a difference of -21.7 ± 13.2 minutes. The 90% confidence interval for the mean difference was equal to -43.7 and +0.3 minutes, was out of the 10% equivalence region (-17.2 and +17.2 minutes), and therefore, estimates were considered not equivalent. Equivalence was only supported for a 30% region (-51.5 to +51.5 minutes) (Table 2). Aggregated estimates for Saturday and Sunday are provided in Fig 4.

#### Section III: Sedentary Time

**Fig 5:**
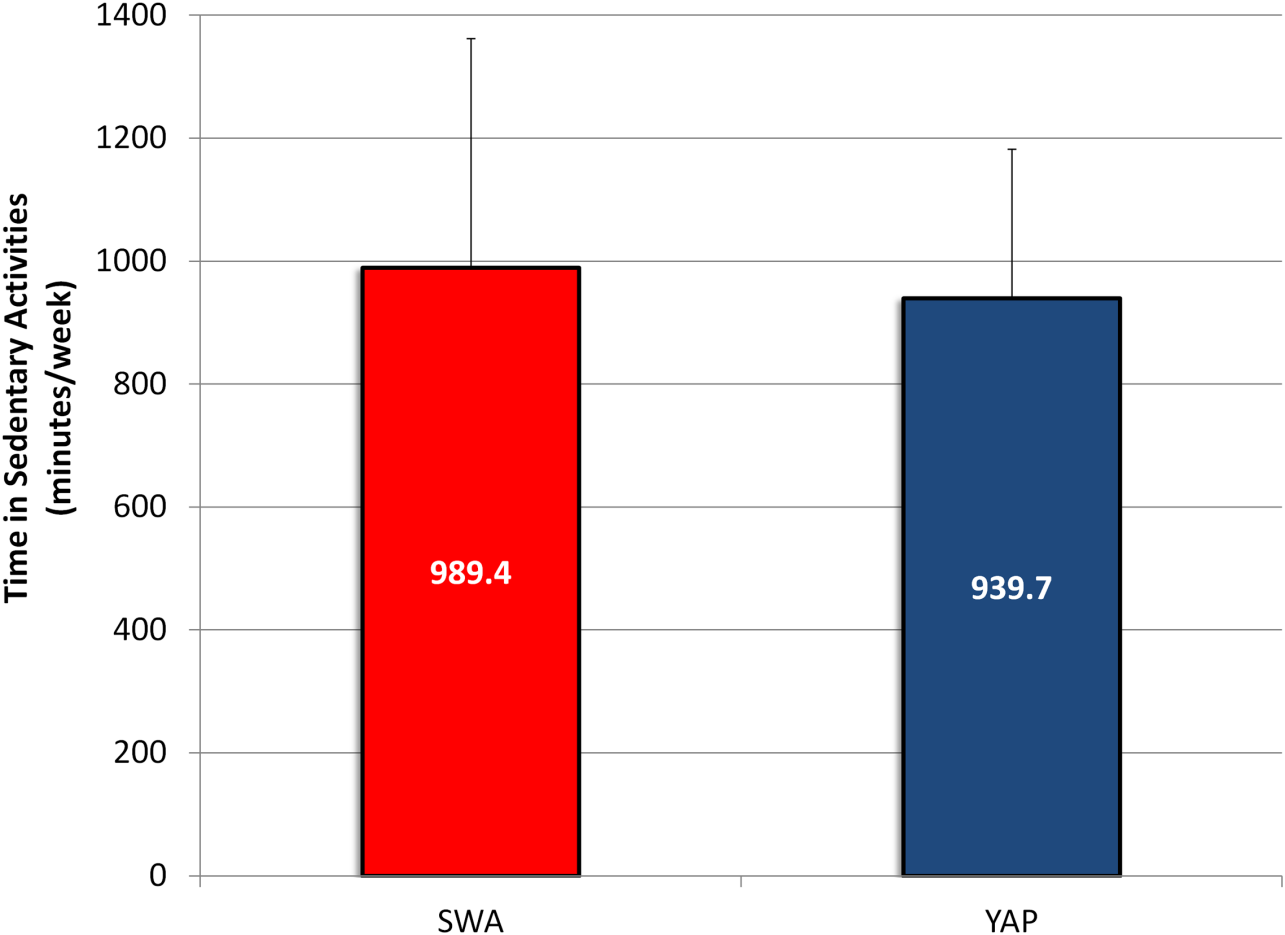
Average estimates and respective standard deviations of recorded and predicted weekly sedentary time at out-of-school.

SWA and YAP estimates of sedentary time were strongly correlated (r (116) = .75, p < .001) (Fig 1 –Panel D) and individual-level error ranged from -54% to +44% of SWA measured sedentary time (Table 2). Average objectively measured and YAP predicted sedentary time during out-of-school on weekdays were 989.4 ± 372.7 minutes and 939.7 ± 242.3 minutes, respectively (difference = -49.7 ± 23.1 minutes). The 90% confidence interval for the mean difference was -88.0 and -11.4 and was within the 10% equivalence region (-98.9 to +98.9 minutes). The estimates obtained from the two measures were considered equivalent (Table 2 & Fig 5) (S4 File).

## Discussion

This study provides preliminary calibration results for a promising self-report tool developed to quantify MVPA and sedentary time in youth from 4^th^-12^th^ grades. The Youth Activity Profile was developed to quantify activity and sedentary levels in specific periods of the week. The majority of the items were found to be significant predictors of recorded activity after controlling for age and gender (exceptions were for the periods capturing lunch, before school and Sunday).

The agreement between predicted and measured activity at the individual-level was poor to moderate and varied by YAP sections. The Limits of Agreement were particularly wide for out-of-school time and well above 100% for Weekend estimates. The error was within -87% to +67% for school-related activity. These differences are likely explained by both the structured nature and length (i.e., fewer minutes) of the school periods being recalled when compared to out-of-school time. We also found that agreement at this level was particularly improved for sedentary estimates. This finding was particularly interesting and can likely be explained by the fact that sedentary behaviors tend to be more stable when compared to active behaviors and therefore, this can facilitate the recall process.

At the group-level, YAP school and out-of-school activity were within 15% and 20% of SWA measured activity values for the respective segments. Both 15% and 20% equivalence regions were tested using established procedures described by the Federal Drug Administration (FDA) in the U.S. [31] and thus, revealed acceptable agreement between the two measures. The calibration models for the weekend section did not perform well and predicted activity obtained from the two related items was only within 30% of SWA values. This level of imprecision illustrates the challenges when assessing activity accumulating during the weekend. Self-report tools elicit recall skills that are more challenged when individuals have to recall longer periods of time. The total amount of time to be recalled was greater for the weekend segment (total of 1800 minutes) so this likely led to more error in estimates. Interestingly, the Saturday item was a significant predictor of activity while the Sunday item β-weight was non-significant. Measured activity on Saturdays and Sundays were very similar however, activity patterns on Sunday might be harder to recall since it relies more on unstructured activity. Structured activity, such as participation in sports most likely occur on Saturdays and therefore, might be easier to recall. In other words, if physical activity does not provide a meaningful stimuli to an individual, the behavior will likely not capture the individual’s attention and therefore will not be stored in memory for future recall [32,33]. Similar to the individual level agreement, estimates from the sedentary section approximated measured sedentary time from the SWA. Predicted values had an average error of 49.7 minutes and were within 10% of SWA values.

To interpret these findings it is important to further emphasize the distinctions between individual- and group-level agreements. These combined can provide a more robust examination of agreement than commonly used correlations that are known to be of limited use [34]. There are inherent challenges in establishing both individual- and group-level agreement for most assessment instruments but researchers rarely distinguish between the two. Individual-level agreement reflects the accuracy of a measure for a single individual or child in the context of this study. One particular useful indicator of individual agreement is the use of Limits of Agreement as proposed by Bland & Altman [29]. This approach provides a good indicator of individual-level agreement since its computed based on the absolute deviation for each individual pair of observations (i.e., criterion vs. calibrated measure) while maintaining the original scale (i.e., computing the standard deviation). This approach has been widely used [34] however, it has also generated some controversy in the field of sports science [35]. Most PA assessments (including objective activity monitors) are “imprecise” at this level as evidenced by relatively large confidence interval for calibrated estimates. Even heart-rate lacks accuracy when identifying bouts of activity at the individual-level; however, the bias (i.e., average error) associated with this instrument can be considered acceptable [36,37]. The same is true for the YAP which exhibited poor individual agreement but good group estimates with aggregated estimates within 10% to 20% of SWA values (with the exception of out-of-school activity). Thus, this level of agreement demonstrates the potential of this tool to assess activity levels in groups of youth (e.g. providing estimates for school-aged students by class or grade). Individual-level agreement can certainly improve the ability of a tool to estimate activity however, lack of individual agreement should not rule out the possibility that instruments can characterize activity in groups.

The concept of group-level agreement is closely related with the accuracy of a measure and implies that estimates obtained from a certain tool when averaged across different individuals provide a good representation of activity level for that group. This level of accuracy can be achieved even though the activity level from each individual composing this group is rather imprecise (e.g., underestimated or overestimated). Similarly to individual-level agreement, validation studies tend to provide a limited examination of agreement at the group-level. Studies often test the hypothesis of no mean differences and force the two measures to be identical or not different than 0 (e.g., paired t-tests). This pre-requisite for validation is very conservative and can be considered naïve given that the two measures are likely to differ to some extent and therefore, it becomes very challenging to not reject the null hypothesis of no mean differences. This test of mean differences was identified by Zaki and colleagues as the most popular group-level assessment technique among validation studies in medical research [34]. Additionally, testing this hypothesis also penalizes validation studies that have larger sample sizes and therefore, more statistical power to detect small differences. The issue of relying on p-values and their magnitude has long been discussed, and dates to at least the 70’s and 80’s [38,39,40]. It is often identified as favoring the purpose of the study by detecting statistically significant effects that are often not deemed meaningful and only result from increasing the sample size [41,42,43]. In validation studies it is particularly problematic and experts have recommended alternative designs to address this limitation [34].

We have overcome this classic limitation by using bioequivalence testing with the two one-sided t test (TOST) [30,44]. This procedure allows the two measures to differ by a predetermined but arbitrary range at a 90% confidence interval. In the current study it was used to determine if self-report estimates obtained from the YAP were within 10% of accelerometer values but the 10% range is rather tight and somewhat conservative since less precision may still be acceptable in many situations. The definition of equivalence is rather fundamental when using this approach [44] and is likely to vary depending on the application of the tool being validated. We have therefore, established different zones of equivalence when 10% equivalence could not be supported (i.e., 15%, 20%, and 30%). Thus, physical activity researchers, that rely on self-reports will clearly benefit if they know that activity estimates obtained from the self-report tool are expected to fall within 10% to 20% of what they would be if objective measures were used instead.

In addition to this more appropriate test, a strength of our approach was the use of a robust design and analytical plan [16]. There are four design features that specifically helped to ensure more generalizable predictions: 1) Replicate data were obtained from 343 youth aged 9–18 years across different seasons and subject to advanced data reduction procedures to ensure high quality of the data; 2) Data were collected in groups of 15–20 and each participant had to go through a recall quality check in order to ensure that participants were appropriately using contextual information elicited by YAP items; 3) The study design used a counterbalanced order of data collection among participants of different ages and at different seasons to control for potential age and seasonal effects and to also capture natural variability; 4) Detailed school schedule information was collected from each school involved in the study and therefore, we were able to determine each participant’s daily schedule over a two week period. The complexity of this task cannot be underestimated since the school curriculum (e.g. Physical education, recess) in the U.S. is very diverse. Late starts and early outs from school were taken into account, as well as field trips that occurred during data collection. With this approach we were able to accurately capture and define each of the important windows of time. Follow-up analyses indicated that we were able to capture 94% of the total MVPA that occurred between approximately 7:00AM and 10:00PM. This precision was needed for the calibration but the advantage of the methodology is that estimates can be obtained for other schools and times using the resulting generalized prediction equations.

The calibrated YAP tool can have important applications since it enables the subjective data from the YAP to produce estimates that match those that would be obtained from objective monitors. A good example of the potential of estimated weekly activity is the ability to identify the percentage of children that meet current physical activity guidelines (e.g. 60 minutes a day) [45]. Supplemental analyses were conducted to directly compare the ability of the YAP to identify individuals that met current physical activity guidelines (using the objective SWA data as the criterion). The aggregated minutes obtained from the YAP items identified 70% of children not meeting recommended guidelines (Sensitivity = 0.70). Additionally, 69.2% of individuals meeting current guidelines were also correctly identified by YAP estimates (Specificity = 0.69). These indicators are similar to what we found in our previous work with the PAQ [11]. A similar approach was used to examine the ability of school-related YAP items to identify children that meet 30 minutes of daily activity during school time. The Institute of Medicine (IOM) has recommended that schools should provide opportunities for youth to accumulate at least half of the daily recommended physical activity (e.g. 30 minutes) [46]. Sensitivity and Specificity associated with this cutpoint were equal to 0.76 and 0.61, respectively. These estimates are very reasonable and provide schools the ability to determine compliance with recommended IOM activity levels.

We acknowledge that the calibration algorithms reported in the study were developed in the same group of individuals that were used for calibration. Separate sets of weekly activity data were used but it is possible that this enhanced agreement. Therefore, the algorithms obtained from this study need to be tested and, if needed, refined on an independent sample of youth.

## Conclusions

In conclusion, the YAP can accurately estimate activity levels in groups of individuals. The utility of the YAP can be improved if additional work is done to refine items that were shown to have limited predictive ability of recorded activity. Results support our research hypothesis and show that the YAP can be used to accurately estimate activity at the group-level in different contexts (school and out-of-school settings). Additionally, this self-report tool can also provide accurate estimates of group-level sedentary time. We anticipate that more items can be included in order to obtain sedentary time accumulated during weekend days. The YAP shows potential and can be broadly disseminated and also used in educational settings as part of coordinated physical education assessments. Children can learn about their activity while completing the assessment and the aggregate data provide a valid indicator of group level data.

## Supporting Information

S1 File Computation of MVPA using YAP scores. (DOCX)

S2 File Flow of Participants. (DOCX)

S3 File Preliminary Validation. (DOCX)

S4 File Error Analysis. (DOCX)
