# Supplementary material for: Validity and Calibration of the Youth Activity Profile
Source: PLoS One. 2015 Dec 2;10(12):e0143949. doi: 10.1371/journal.pone.0143949 (PMC4668067; doi:10.1371/journal.pone.0143949)
Supplement: S1 File — (DOCX) [file pone.0143949.s001.docx]

**Computation of MVPA using YAP scores**

Introduction and Methods

This supplemental document describes how MVPA was computed for the cross-validation phase. These procedures can also be used in future applications of the YAP when predicting MVPA time.

Predicted scores were converted into weekly minutes of activity/sedentary following the five steps below:

1. The predicted percent MVPA score was multiplied by the respective segment wear time (e.g. Recess activity = Predicted daily percent MVPA during recess X Recess wear time).
2. Estimated minutes of activity per segment were multiplied by 5 if obtained from: transportation to/from school, recess, or lunch and by 2 if obtained from PE item. These estimates were aggregated in order to reflect In-School Activity accumulated during a full week (5 days);
3. Estimated minutes of activity were multiplied by 5 if obtained from: before-school, after-school, or evening items. These estimates were aggregated in order to reflect Out-of-School activity accumulated during a full week;
4. Estimated minutes of activity were multiplied by 1 if obtained from Saturday and Sunday items. These items were aggregated in order to reflect Weekend Activity accumulated during the week;
5. Estimated minutes obtained from the sedentary algorithm were multiplied by 5 in order to reflect minutes of sedentary time accumulated during Out-of-School time. These estimates were used independently.

Steps 2 through 4 relate to sections I and II of the YAP while estimates obtained in step 5 related to section III of the YAP.
