# Supplementary material for: Validity and Calibration of the Youth Activity Profile
Source: PLoS One. 2015 Dec 2;10(12):e0143949. doi: 10.1371/journal.pone.0143949 (PMC4668067; doi:10.1371/journal.pone.0143949)
Supplement: S2 File — (DOCX) [file pone.0143949.s002.docx]

**Flow of Participants**

INTRODUCTION AND METHODS

This supplemental document provides additional detail regarding sample size and flow of participants as data were first screened for quality and representativeness, and later, stratified for calibration and validation. When stratified by week of assessment, there were 252 participants with valid data for at least one segment of activity in week 1 and 221 participants with valid data for week 2 (Figure S1). Because the analyses examined activity for discrete sample windows it was possible to include participant’s data for specific segments. The weekend segment has the lowest compliance rates and resulted in 132 and 134 valid observations for Saturday and Sunday, respectively, while Lunch and Transportation from School had the highest compliance rates with 263 and 268 valid observations, respectively. Average indicators of compliance were similar among the two weeks of data collection.


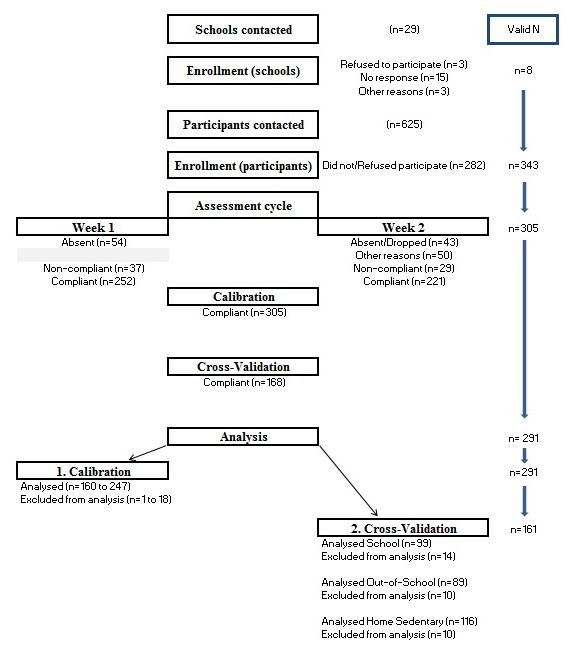


Figure S1. Flow chart of participants.
