# Supplementary material for: Validity and Calibration of the Youth Activity Profile
Source: PLoS One. 2015 Dec 2;10(12):e0143949. doi: 10.1371/journal.pone.0143949 (PMC4668067; doi:10.1371/journal.pone.0143949)
Supplement: S3 File — (DOCX) [file pone.0143949.s003.docx]

**Preliminary Validation**

Introduction & Methods

This supplemental document describes the preliminary validity of each individual YAP item. Once data from SWA was matched with each item we first examined the overall relationship between the observed and the reported data. These relations were examined using Spearman correlations and determined the extent to what each YAP item and respective accelerometer segment ranked activity similarly.

Results

Spearman correlations between each pair of recorded and reported activity ranged from .01 to .24 for school segments and .02 to .33 for out-of-school segments. The ability to rank activity similarly was higher, during the transportation to school (r_s_ (207) = .24, p < .001) and after-school periods (r_s_ (242) = .33, p < .001) and there were no associations found for lunch (r_s_ (225) = .01, p = .90) and before school time (r_s_ (195) =.01, p = .86) activity. Combined percent time in sedentary behavior recorded during the after-school and evening segments was moderately correlated with the YAP sedentary composite score (r_s_ (190) = .47, p < .001). When examined separately, correlations with each sedentary item ranged from .02 to .46. Reported videogame had the lowest association with percent sedentary time (r_s_ (190) = .02, p = .78) while reported cell phone use had the strongest association with this indicator (r (190) = .46, p < .001).
