# Supplementary material for: Validity and Calibration of the Youth Activity Profile
Source: PLoS One. 2015 Dec 2;10(12):e0143949. doi: 10.1371/journal.pone.0143949 (PMC4668067; doi:10.1371/journal.pone.0143949)
Supplement: S4 File — (DOCX) [file pone.0143949.s004.docx]

**Error Analysis**

INTRODUCTION & Methods

This supplemental document describes 1) the methods used to examine the robustness of the statistical models developed during calibration and 2) the methods used to determine differential error associated with the calibration models. Results for each of the analyses are also reported in this file.

**During calibration**, the robustness of the calibration models were examined by inspecting any possible associations with relevant covariates as detected by Pearson correlations greater than .30. These included: age, gender, weight status (based on standard classification categories from the Centers for Disease Control and Prevention), group of assessment, date completed (i.e., completion study date), month of assessment (e.g. December was coded as 12), season (i.e. Winter or Non-Winter season), total number of valid activity segments, average accelerometer wear time, and accelerometer percent day wear. Additional checks included visual examination of heteroscedascity, normality of residuals, and computation of root mean square error (RMSE).

**During cross-validation**, the assessment of differential error was examined as follows. 1) We first examined if absolute residuals were equally distributed across measured minutes of MVPA obtained from the SWA and using Pearson correlations. 2) These analyses were followed by two-way ANOVAs with residual scores (MVPA YAP – MVPA SWA) as the dependent variable and age group and gender as independent variables. Non-differential error is evident if error was not associated with any of these selected factors. Adjusted means were significantly different than 0 if p was < .01.

results

**Calibration (School Activity):** Upon examination of residuals for each model, there was no evidence that residuals were associated with covariates of interest (e.g., BMI percentile, date completed, wear time, etc) for all the items except for recess. Date completion of the study was positively correlated with residuals computed from this period (r (160) = .34, p < .001). This indicated that error was higher for data collected later in the year (e.g. December). There was also no evidence of heteroscedasticity, and residuals were normally distributed for all the items suggesting that error was homogeneously distributed across different levels of activity accumulated at school. Average error as indicated by the root mean square error (RMSE) ranged between 8.9% (Lunch) and 28.9% MVPA (PE). In other words, %MVPA could be estimated during PE with an average error of 28.9 units (defined in % time).

**Calibration (Out-of-School Activity):** Upon examination of residuals for each model, there was no evidence that residuals were associated with relevant covariates (e.g., BMI percentile, date completed, wear time, etc). There was also no evidence of heteroscedasticity, and residuals were normally distributed. Average error (RMSE) ranged between 4.9% (Sunday) and 10.3% (After-School).

**Calibration (Sedentary Time):** There was no evidence of differential error, heteroscedascity, or violations of normality. The RMSE for this section was 12.3%.

**Cross-Validation (School Activity):** The examination of differential error revealed that residuals were not associated with measured activity levels (r (99) = .13, p = .20) but the ANOVA results revealed that error varied by age group (F (2, 95) = 10.85, p < .001). Results from the post-hoc analysis showed that mean error in high school participants was of greater magnitude and equal to -55.3 ± 10.4 minutes (p < .001). This error was considerably lower for elementary school participants (2.2 ± 7.9) and middle school participants (1.1 ± 14.1), respectively (Figure S2.1). There was no significant effect for gender (F (1, 95) = 0.08, p = .77).

**Cross-Validation (Out-of-School Weekday Activity):** The correlation between absolute residuals and SWA activity was not significant (r (90) = .20, p = .06) and the effect of age group on bias was borderline significant (F (2, 86) = 3.10, p = .05). Error was considerably higher in middle school participants (104.5 ± 44.5 minutes of MVPA) than values for elementary school participants (-14.6 ± 21.1) and high school participants (-16.1 ± 31.7), respectively (Figure S2.2). Error was similar among boys and girls (F (2, 86) = 1.35, p = .25).

**Cross-Validation (Out-of-School Weekend Activity):** Absolute error obtained from this segment was positively correlated with measured activity scores (r (66) = .55, p < .001) indicating that error was larger in more active individuals. A closer examination of the direction of the error indicated that activity was underestimated at higher activity level scores and overestimated for lower activity levels. There was no further evidence of differential error among age groups (F (2, 62) = 2.60, p = .08) (Figure S2.3). The magnitude of error was similar among boys and girls (F (1, 62) = 1.90, p = .17).

**Cross-Validation (Sedentary Time):** Absolute residuals were not correlated with SWA activity estimates (r (116) =.13, p = .17); however, error was significantly different among age groups (F (2, 112) = 8.42, p < .001) and considerably higher among middle school participants (Mean = -209.0 ± 52.0 minutes) (Figure S2.4). There was no gender effect on error (F (1, 112) = 3.64, p = .06).


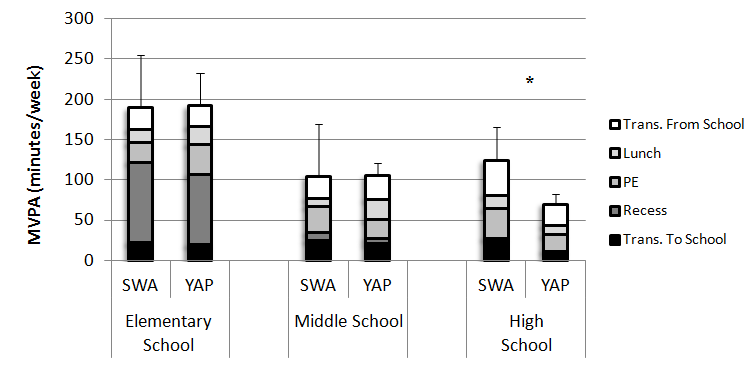


Figure S2.1. Average SWA and YAP estimates and respective standard deviations, of weekly MVPA for elementary, middle and high school participants. Estimates were segmented by school activity windows.

* Significantly different with p < .01.


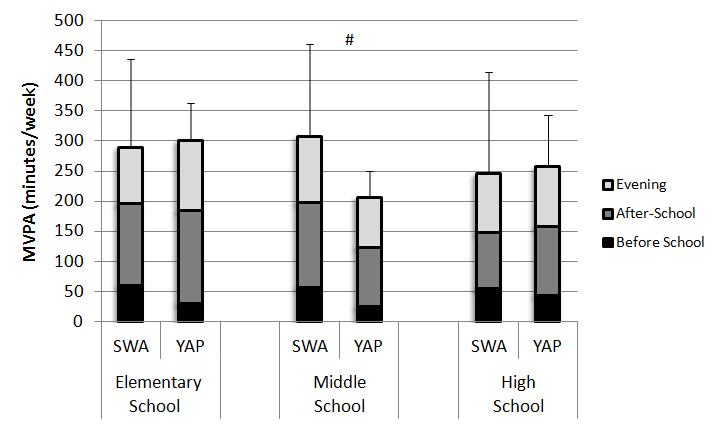


Figure S2.2. Average SWA and YAP estimates and respective standard deviations of weekly Out-of-School MVPA for elementary, middle and high school participants. Estimates were segmented by respective activity windows. # Borderline significant with p < .05.


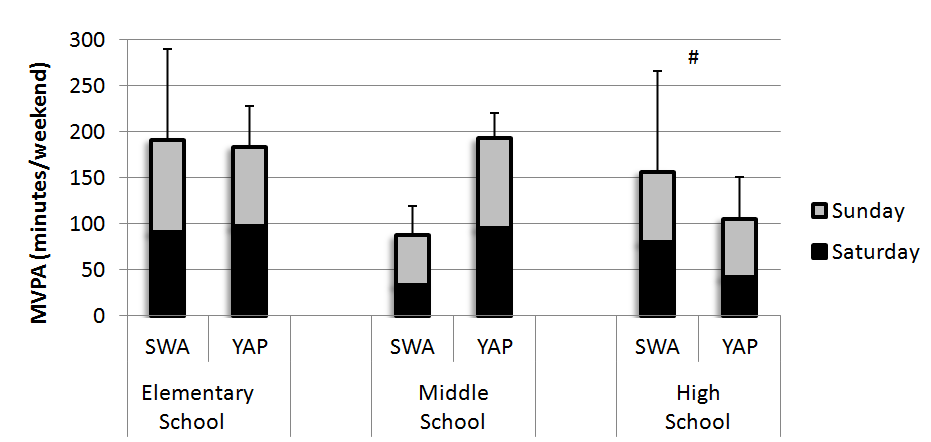


Figure S2.3. Average SWA and YAP estimates and respective standard deviations of weekend MVPA for elementary, middle and high school participants. Estimates were segmented by respective activity windows. # Borderline significant with p < .05.


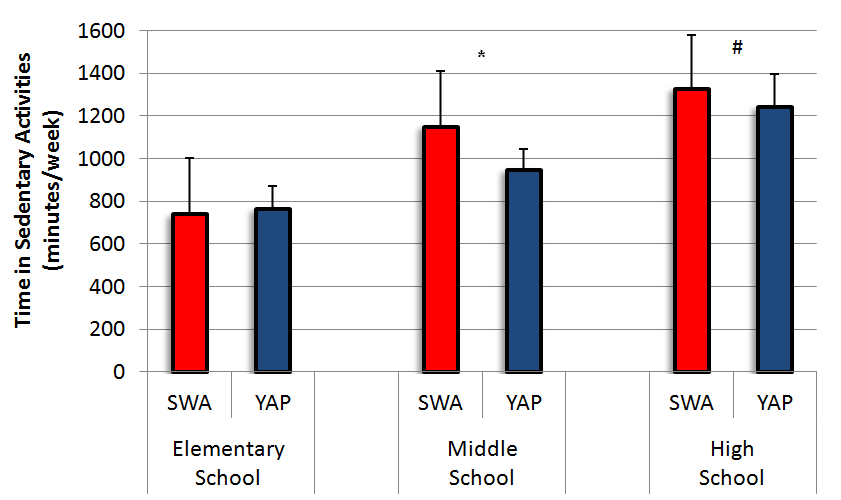


Figure S2.4. Average SWA and YAP estimates and respective standard deviations of sedentary activity for elementary, middle and high school participants. * Significantly different with p < .01). # Borderline significant with p < .05.
